# From X-ray crystallographic structure to intrinsic thermodynamics of protein–ligand binding using carbonic anhydrase isozymes as a model system

**DOI:** 10.1107/S2052252524004627

**Published:** 2024-06-10

**Authors:** Vaida Paketurytė-Latvė, Alexey Smirnov, Elena Manakova, Lina Baranauskiene, Vytautas Petrauskas, Asta Zubrienė, Jurgita Matulienė, Virginija Dudutienė, Edita Čapkauskaitė, Audrius Zakšauskas, Janis Leitans, Saulius Gražulis, Kaspars Tars, Daumantas Matulis

**Affiliations:** a Department of Biothermodynamics and Drug Design, Institute of Biotechnology, Life Sciences Center, Vilnius University, Saulėtekio 7, 10257 Vilnius, Lithuania; b Department of Protein - DNA Interactions, Institute of Biotechnology, Life Sciences Center, Vilnius University, Saulėtekio 7, 10257 Vilnius, Lithuania; c Latvian Biomedical Research and Study Centre, Ratsupites 1 k-1, 1067 Riga, Latvia; d Sector of Crystallography and Chemical Informatics, Institute of Biotechnology, Life Sciences Center, Vilnius University, Saulėtekio 7, 10257 Vilnius, Lithuania; Chinese Academy of Sciences, China

**Keywords:** drug discovery, protein structure, molecular recognition, X-ray crystallography, intermolecular interactions, carbonic anhydrase isozymes, protein–ligand binding, intrinsic thermodynamics, binding assays

## Abstract

Carbonic anhydrase (CA) was among the first proteins whose X-ray crystal structure was solved to atomic resolution. CA proteins have essentially the same fold and similar active centers that differ in only several amino acids. Primary sulfonamides are well defined, strong and specific binders of CA. However, minor variations in chemical structure can significantly alter their binding properties. Over 1000 sulfonamides have been designed, synthesized and evaluated to understand the correlations between the structure and thermodynamics of their binding to the human CA isozyme family. Compound binding was determined by several binding assays: fluorescence-based thermal shift assay, stopped-flow enzyme activity inhibition assay, isothermal titration calorimetry and competition assay for enzyme expressed on cancer cell surfaces. All assays have advantages and limitations but are necessary for deeper characterization of these protein–ligand interactions. Here, the concept and importance of intrinsic binding thermodynamics is emphasized and the role of structure–thermodynamics correlations for the novel inhibitors of CA IX is discussed – an isozyme that is overexpressed in solid hypoxic tumors, and thus these inhibitors may serve as anticancer drugs. The abundant structural and thermodynamic data are assembled into the Protein–Ligand Binding Database to understand general protein–ligand recognition principles that could be used in drug discovery.

## Abstract 2

Rational drug discovery and design require a deep understanding of the structure and thermodynamics of protein–ligand interactions. Here, the human carbonic anhydrase family of enzymes and their specific sulfonamide ligands are used to describe binding assays and crystal structures for understanding of protein–compound recognition principles.

## 1. Introduction

Structural biology plays a central role in drug discovery (Renaud *et al.*, 2016 ▸; Nasim & Qureshi, 2022 ▸). Small-molecular-weight organic compounds could be biologically active due to binding to proteins and other macromolecules. Such compounds are often found in nature or could be rationally designed and synthesized. According to the drug-discovery paradigm, the first hypothesis points to a protein involved in a disease progression (Huang *et al.*, 2012 ▸; Liu *et al.*, 2022 ▸) and an assumption that an inhibitor could alter the disease. A compound is then designed to bind the protein and possibly inhibit or activate its enzymatic action (Minetti & Remeta, 2022 ▸). A crucial step to understand the mode of ligand binding and further optimize the interaction is to determine the atomic resolution structure of the protein in complex with the bound compound. This identifies the mode of interaction and the functional groups that determine the affinity of the compound binding to the protein target (Timothy & Sanjeev, 2007 ▸; Maveyraud & Mourey, 2020 ▸). Although the complex structure is usually determined by X-ray crystallography, this method does not directly provide information on compound affinity (Schlichting, 2005 ▸; Danley, 2006 ▸). Biophysical assays are necessary to determine thermodynamic and kinetic parameters of protein–ligand interaction.

Carbon dioxide is a cellular metabolism product that must be eliminated from the body. Due to the limited carbon dioxide solubility, bicarbonate serves as the primary mode of CO_2_ transport in the blood (Alka & Casey, 2014 ▸; Occhipinti & Boron, 2019 ▸). Therefore, rapid conversion between these two forms is critical (Casey, 2006 ▸). Although these conversions can occur spontaneously, their natural rate is insufficient to meet the needs of an organism. The discovery of the catalyst responsible for the reaction between carbon dioxide and bicarbonate came with the isolation of carbonic anhydrase (CA, EC4.2.1.1.) from bovine blood in 1932 (Meldrum & Roughton, 1933 ▸). This enzyme is essential for accelerating the conversion of carbon dioxide to bicarbonate ions and protons, increasing the rate up to a million-fold.

It took about 40 years of CA research before Liljas *et al.* (1972 ▸) determined the first crystal structure of CA II, now considered a model protein, to a resolution of 2.0 Å. This and later crystallographic studies revealed that the CA II molecule has an ellipsoidal shape, measuring ≈40 Å × 40 Å × 40 Å. The cone-shaped active site has a depth of about 15 Å, with the catalytic zinc located at the base and coordinated by three histidine residues. The enzyme core comprises a ten-stranded β sheet surrounded by seven right-handed α helices. The active site of CA II has two sides of different polarity: one with hydrophobic residues (Ile91, Val121, Phe131, Val135, Leu141, Val143, Leu198, Pro202, Leu204, Val207 and Trp209) and the other with hydrophilic residues (Asn62, His64, Asn67, Gln92, Thr199 and Thr200). The hydrophobic side hosts the substrate CO_2_ binding site and the hydrophilic residues help stabilize the ordered water network essential for proton transfer (Lomelino *et al.*, 2018 ▸).

CA occurs in various structural forms in different organisms. To date, researchers have identified eight structural classes of CAs, designated by Greek letters: α, β, γ, δ, ζ, η, θ and ι (Jensen *et al.*, 2019 ▸). Humans have 15 α-CA isozymes that regulate pH and fluid balance, and they are involved in various physiological and pathological processes (Imtaiyaz Hassan *et al.*, 2013 ▸; Frost, 2014 ▸). The 15 isozymes differ in tissue distribution and have various catalytic activities and cellular localization. However, they share a similar overall structure, with only minor differences in their active site (Lomelino *et al.*, 2018 ▸). Several human CA isozymes are recognized as therapeutic targets in glaucoma, edema, epilepsy, obesity and cancer (Ciccone *et al.*, 2021 ▸; Mboge *et al.*, 2018 ▸; Lomelino & McKenna, 2016 ▸), but the design of selective inhibitors for specific isozymes remains challenging due to the high structural similarity.

The first-generation drug to treat/control disease using CA inhibition was acetazolamide, which entered medical use in 1953 and is still used as a systemic medication for glaucoma, altitude sickness, epilepsy, idiopathic intracranial hypertension and other conditions (Rankin, 2007 ▸; Scott & Njardarson, 2018 ▸). Over the next 70 years, more than 20 CA inhibitors entered clinical practice. However, none of them show significant CA isozyme selectivity. Off-target binding can lead to undesired effects since CAs participate in crucial metabolic processes. Furthermore, off-target binding decreases the available drug fraction, thus necessitating a higher dose. Although many chemical classes of compounds have been shown to inhibit CA activity (Lomelino *et al.*, 2016 ▸; Singh *et al.*, 2018 ▸; Kumar *et al.*, 2021 ▸), all clinically used CA inhibitors have either a sulfonamide or its bioisostere group that directly interacts with the catalytic zinc and displaces the zinc-bound water molecule (Swenson, 2014 ▸).

In this study, we describe thermodynamic analysis of CA IX-selective compounds binding to their target and off-target isozymes. CA IX is the CA isozyme that has direct relevance to cancer – its expression is induced in hypoxia under the control of HIF-1 (Pastorek *et al.*, 1994 ▸; Saarnio *et al.*, 1998 ▸). In tumors, CA IX helps cancer cells survive under hypoxic conditions, while in healthy tissues, its expression is limited to the gastrointestinal tract (Becker, 2020 ▸).

Numerous assays may be applied to measure protein–ligand binding interactions, described for CA II isozyme as a model protein (Krishnamurthy *et al.*, 2008 ▸). We primarily use fluorescence-based thermal-shift assay (TSA or FTSA) and consider it the most robust and accurate method to determine the *K*_d_ of protein–ligand interactions because it uniformly covers a wide range from millimolar to picomolar affinities. We described TSA previously and discussed its advantages and limitations over the other orthogonal binding assays (Matulis *et al.*, 2005 ▸; Cimmperman *et al.*, 2008 ▸; Petrauskas *et al.*, 2024 ▸). We have made a web-based service called *Thermott* that analyses user-supplied thermal-shift data, determines the binding parameters, and thus simplifies TSA data analysis (Gedgaudas *et al.*, 2022 ▸). In the literature, enzyme activity inhibition is the primary assay to describe sulfonamide binding to CA isozymes. However, this assay has limitations that prevent the discovery of tight picomolar inhibitors, as discussed below.

**Figure 1:**
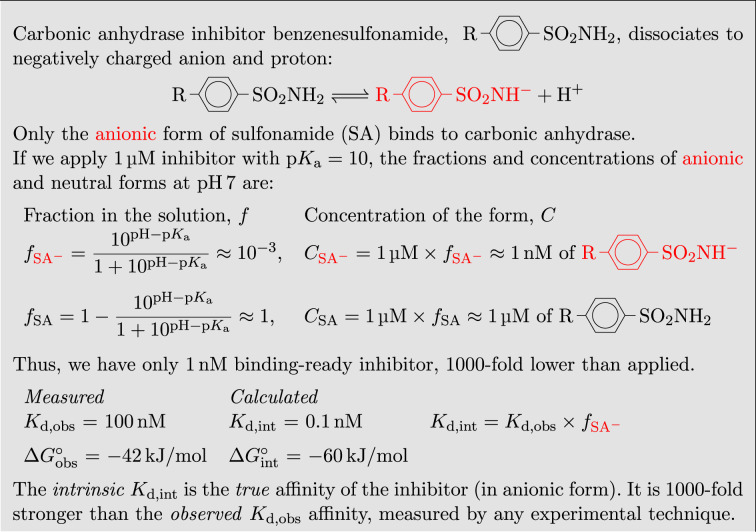
The concept of *intrinsic* and *observed* dissociation constant explained using benzenesulfonamide deprotonization reaction and fractions of binding-ready species.

Protein–ligand interactions often occur simultaneously with the linked protonation–deprotonation reactions (Bahr *et al.*, 2023 ▸; Huang *et al.*, 2012 ▸; Hognon *et al.*, 2022 ▸). Such reactions hide the actual thermodynamic parameters of interaction and may be misinterpreted when studying the underlying forces in the protein–ligand complex (Bradshaw & Waksman, 1998 ▸). To dissect binding-energy contributions from these linked protonation–deprotonation reactions, we emphasize the importance of the intrinsic energetics. Fig. 1 ▸ shows a simplified concept to understand why systematic errors often occur in the thermodynamic interpretation of the binding data.

The example in Fig. 1 ▸ shows that any experimental technique will determine the *K*_d_ (dissociation constant) equal to 100 nM, a relatively weak affinity for the drug candidate. However, the user incorrectly assumed that the entire 1 µM of inhibitor, applied in the assay, participated in the reaction. In reality, only 1 nM of the compound was in the binding-ready form. If the concentration of the binding-ready form was 1 µM, then the *K*_d_ would be 0.1 nM and the inhibitor would be considered as a high-affinity inhibitor of the enzyme. Only the intrinsic values should be used for structure–thermodynamics correlations (Gomez & Freire, 1997 ▸; Krishnamurthy *et al.*, 2007 ▸, 2008 ▸).

The example in Fig. 1 ▸ is simplified, excluding the effect of protein protonation on the observed binding parameters. It was previously shown by applying biophysical techniques that the binding of sulfonamides to CA isozymes has a U-shape dependence on pH. Later, by applying neutron-diffraction crystallography (Fisher *et al.*, 2012 ▸), it was confirmed that sulfonamide binds in deprotonated anionic form to the Zn-bound water form of CA. The p*K*_a_ of the Zn-bound water in the active site of 12 human CA isozymes is between 6 and 7, and only the p*K*_a_s of CA I and CA XIII are 8 (Linkuvienė *et al.*, 2018*b* ▸). Thus, the majority fraction of CA I and CA XIII is in its protonated form, capable of binding the deprotonated sulfonamide. In contrast, benzenesulfonamides have p*K*_a_s between 8 and 11. Therefore, at pH 7 only a small fraction is in the anionic form, capable of binding CA, and the observed affinity values mostly depend on the sulfonamide protonation.

To make it easier to analyze abundant data scattered among numerous publications, we assembled a database of the X-ray crystal structures of CA–ligand complexes and the intrinsic thermodynamics of binding (Lingė *et al.*, 2023 ▸). It is the only database that systematically analyzes intrinsic thermodynamics and kinetics of protein–ligand binding. The freely accessible database could be used when analyzing protein–ligand recognition principles and applied for *in silico* drug discovery.

In this article, we performed correlation analysis between several new crystal structures and the thermodynamics of binding of two groups of compounds, fluorinated (VD series) and chlorinated (EA series) benzenesulfonamides. Their binding to 12 human CA isozymes was studied with the goal to determine the factors that are important for the affinity and selectivity towards particular CA isozymes, especially the cancer-associated CA IX.

## 2. Results

### 2.1. X-ray crystal structures of sulfonamide binding to carbonic anhydrases

Hundreds of X-ray crystallographic structures of free or inhibitor-bound CAs from humans and other organisms have been solved at atomic resolution. The main class of CA inhibitors is the compounds bearing the primary sulfonamide functional group. Such compounds bind to CA isozymes by making a coordination bond between the negatively charged sulfonamide amino group nitrogen and the Zn^2+^ ion. Our research group has solved 147 X-ray crystal structures of various sulfonamide compounds bound to different human CA isozymes and deposited them in the Protein Data Bank (PDB). The sulfonamide amino group made the coordination bond with the Zn^2+^ ion in all cases. However, the compounds bound the proteins with highly variable affinities, ranging from barely detectable millimolar affinity to extremely high picomolar affinity. In extreme cases, some compounds exhibited billion-fold differences in the value of the dissociation constant *K*_d_. Therefore, despite the relatively similar recognition mode via the coordination bond, the compound’s exact chemical structure significantly influenced the affinity.

Here, we describe CA II and CA IX recognition details of two structurally highly similar sulfonamide compounds, EA3-3 and EA5-3. Crystallization of CA IX is rather challenging and thus we used a mimic of CA IX, called chimeric CA IX (chCA IX). The chimeric protein is a multiple mutant of CA II, where six amino acids in the active site of CA II are replaced by the amino acids present in CA IX at the same positions. We have previously demonstrated that such chimeric protein mimics actual CA IX to a rather good approximation (Dudutienė *et al.*, 2014 ▸).

**Figure 2:**
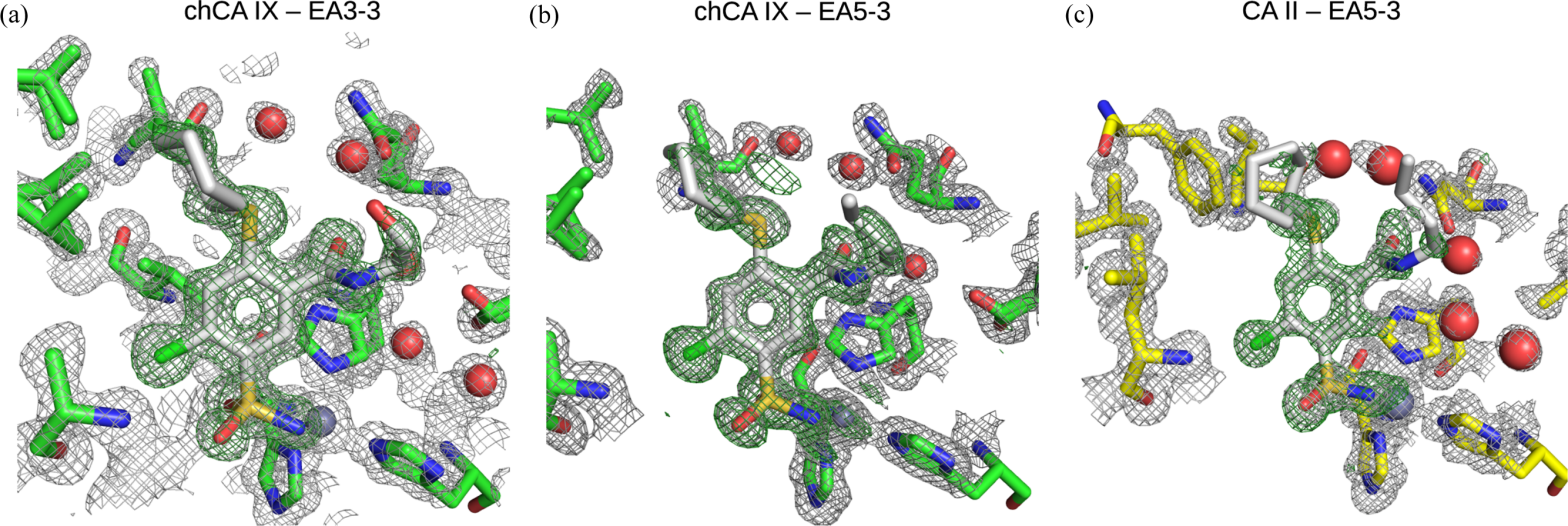
X-ray crystallographic structure determination of inhibitors EA3-3 and EA5-3 binding to CA II and chimeric CA IX (a multiple amino acid mutant of CA II mimicking amino acids of the CA IX active site). The electron density of the inhibitors is shown in green. Amino acids that were mutated in CA II to reproduce the CA IX active site are also shown, as well as histidine residues coordinating the Zn^2+^ ion. Electron-density maps were calculated in the absence of inhibitors. The 2*F*_o_ − *F*_c_ map contoured at 1σ is gray, while the difference map *F*_o_ − *F*_c_ contoured at 3σ is green. Residues of chCA IX are colored green and CA II is yellow. The inhibitors are light gray. The Zn^2+^ ion is shown as a gray sphere and water molecules are shown as red spheres.

Table 1 ▸ shows the refinement statistics of the three crystal structures, while the structural arrangement of the compounds in the active sites and the electron-density maps are shown in Fig. 2 ▸. The sulfonamide amino group formed the coordination bond with the Zn^2+^ ion in all three structures. The orientations of compounds were similar in CA II and chCA IX, but the position of cyclohexane was essential for the recognition of CA IX. The crystal structures provided positions of all atoms in the complex. However, they did not provide information on the affinity or other thermodynamic parameters of binding, which will be described in the following sections.

### 2.2. Assays to measure protein–ligand binding *in vitro*

Numerous techniques could be used to determine protein–ligand binding affinity, or in other words, the change in the standard Gibbs energy upon ligand binding to protein. Other thermodynamic parameters – such as binding enthalpy, entropy, heat capacity, volume and compressibility – may also provide insights into the interaction mechanism. In addition to thermodynamic parameters, the binding kinetics may also be crucial for the understanding of recognition principles, as they show the reaction order, the approach of the on-rate to the diffusion-limited rates, and the stability of the protein–ligand complex (the residence time or off-rate).

To determine the structure–thermodynamics correlations, we primarily perform the TSA, the stopped-flow assay of enzymatic activity inhibition (SFA) and isothermal titration calorimetry (ITC). We consider the TSA as the most robust and easy-to-use assay, spanning the largest dynamic range of affinities among all mentioned assays. TSA provides accurate affinities from millimolar to picomolar *K*_d_. However, TSA does not provide information on whether the compound inhibits the enzymatic activity. The SFA is the most applicable for this purpose, but it spans a lower affinity range than the TSA. ITC is indispensable when we need to determine the enthalpy, entropy and heat capacity of binding.

**Figure 3:**
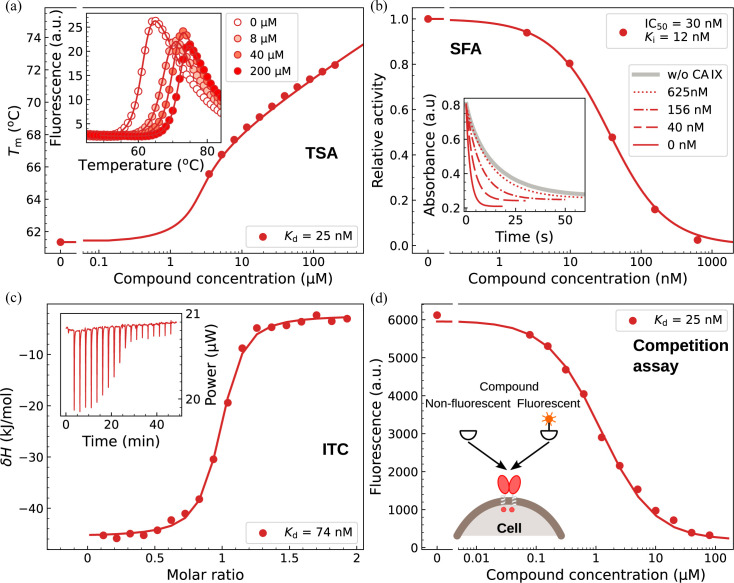
Affinity measurements of benzenesulfonamide-bearing compound VD10-13 binding to CA IX by using four different techniques: (*a*) FTSA, (*b*) SFA of CO_2_ hydration enzymatic activity inhibition, (*c*) ITC and (*d*) competition assay for cell-expressed CA IX. The insets show raw data curves, while the main panels show processed data dosing curves used for dissociation constant (*K*_d_) calculation.

To determine the affinity of sulfonamide binding to CA IX expressed in cancer cells, we developed an assay where we applied the competitive binding model where a fluorescently labeled high-affinity compound competes with an unlabeled one for the same binding site of CA IX. Fig. 3 ▸ shows the determination of fluorinated compound VD10-13 {2,3,5,6-tetrafluoro-4-[(2-hydroxyethyl)thio]benzenesulfonamide} binding to CA IX by using all four methods (TSA, SFA, ITC and the competition assay).

#### 2.2.1. Fluorescence-based thermal-shift assay

The TSA is based on the observation that the bound ligand increases the protein’s melting temperature in a dose-dependent manner. A purified protein has a relatively well defined temperature at which it unfolds and denatures. The addition of a stoichiometric amount of high-affinity ligand stabilizes the complex and increases the melting temperature. However, a tenfold increase in ligand concentration above the stoichiometric ratio will further increase the melting temperature. Therefore, the protein–ligand complex does not possess a well-defined melting temperature (Fukada *et al.*, 1983 ▸; Matulis *et al.*, 2005 ▸).

Fig. 3 ▸(*a*) shows how the affinity of the VD10-13 compound for purified recombinant CA IX was determined by the TSA method. The inset shows the fluorescence dependencies on the temperature at various added compound concentrations. There is a shift of *T*_m_ upon an increase in ligand concentration. There is no need to obtain datapoints at ligand concentrations below the protein concentration. Only the datapoints that determine the shift are important for the *K*_d_ determination. The TSA resultant *K*_d_ was equal to 25 nM.

#### 2.2.2. Assay of inhibition of the enzymatic activity

There are several variations of the enzymatic activity inhibition assay of CAs (Khalifah, 1971 ▸; Colombo & Pinelli, 1981 ▸; Baranauskienė & Matulis, 2019 ▸). CA enzyme catalyzes CO_2_ hydration to bicarbonate and protons, but different reactions, such as hydrolysis of *para*-nitrophenylacetate ester, are often followed due to the assay’s simplicity. However, we prefer an SFA of the inhibition of CA enzymatic activity of CO_2_ hydration (Khalifah, 1971 ▸; Smirnovienė *et al.*, 2017 ▸). This assay is relatively robust and could be used to quantify the *K*_d_ of the studied compound binding to a particular CA isozyme. However, the assay has a significant disadvantage due to a varied limit of *K*_d_ determination for different CA isozymes. This limit depends on the enzyme concentration used in the assay. For example, to measure CA I enzyme activity, we need to use a minimum 0.1 µM of this isozyme. Therefore, the highest-affinity IC_50_ that could be measured at this concentration is 0.1 µM, the same as enzyme concentration. Thus, inhibitor affinities that are stronger than 0.1 µM would show as equal to 0.1 µM. All inhibitors that possess higher affinities would not be discovered. For CA IX, the situation is slightly different because this enzyme has greater specific activity than CA I, and the assay can be performed at 10 nM enzyme concentration. Thus, the IC_50_ limit of determination is around 10 nM, tenfold lower than CA IX. Some equipment and assay optimization allowed enzyme concentration to be further diminished to ≈1 nM and thus reach greater affinities (Kugler *et al.*, 2020 ▸). This difference among CA isozymes may lead to misinterpretation of compound selectivities and affinities.

Fig. 3 ▸(*b*) shows a typical dosing curve and the underlying raw data (in the inset) to determine the IC_50_ of the compound inhibition effect. The value may be transformed to the *K*_i_ inhibition constant if the Michaelis–Menten constant *K*_M_ and the substrate concentration ([S]) are known, by using the Cheng–Prusoff equation: 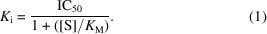
The inhibition constant *K*_i_ is not entirely equivalent to the dissociation constant *K*_d_ but their values could be compared. This assay proves that a compound is an enzyme inhibitor, not just a random protein site binder. Other assays, such as TSA or ITC, cannot make this distinction.

#### 2.2.3. Isothermal titration calorimetry

ITC is often considered a central assay to determine compound affinities for proteins. The main advantage of ITC is that it is the only assay that measures directly the heat effects and, therefore, it determines the enthalpy if heat is measured at a constant pressure. No other assay can do this directly. The enthalpy could also be determined by performing other assays at different temperatures and then calculating the enthalpy by applying the van’t Hoff equation. However, this indirect determination of the enthalpy is much less accurate than direct determination by ITC (Klebe, 2015 ▸).

If the affinity *K*_d_ of a compound is around 0.1 µM to 1 µM, the ITC yields highly accurate *K*_d_ values. However, ITC has significant limitation in the narrow dynamic range of *K*_d_ values. Since the best results are obtained when the Wiseman parameter is within the range from 5 to 500 (Wiseman *et al.*, 1989 ▸; Velazquez-Campoy, 2015 ▸), thus, at 10 µM enzyme concentration required for the assay, the obtainable *K*_d_ range is 2 µM–20 nM. Further range extension is possible only by performing the competition assay with a strong binder (Krainer & Keller, 2015 ▸), complicating the data analysis.

Fig. 3 ▸(*c*) shows a typical dosing curve obtained by ITC, with the raw data curve shown in the inset. The curve yielded a *K*_d_ value of 74 nM. This value is slightly higher but still comparable to the values obtained by other assays. The standard error of all *K*_d_ measurements is approximately equal to twofold in the *K*_d_ value (Paketurytė *et al.*, 2021 ▸). Thus, a value of 74 nM is within a range of 37 nM to 150 nM, while a value of 25 nM is within a range of 12 nM to 50 nM. Therefore, the values of 74 and 25 nM are within the error margin of both determinations.

The ITC assay determines the observed enthalpy. This value for the data in Fig. 3 ▸(*c*) is equal to −45 kJ mol^−1^. However, this value contains linked protonation reactions that could contribute comparable enthalpies due to buffer protonation. Therefore, it is essential to dissect all contributing reaction enthalpies and calculate the intrinsic enthalpy (Linkuvienė *et al.*, 2018*b* ▸).

#### 2.2.4. Competition assay in live cancer cells

When we designed a series of high-affinity compounds for CA IX as potential anticancer agents, it was necessary to demonstrate that these compounds recognize and bind exclusively to CA IX, whose expression increases upon growing cancer-cell cultures under hypoxia. It was essential to demonstrate whether the compounds bind (1) with high affinity, and (2) specifically and selectively to CA IX. For this purpose, we designed a compound GZ19-32 that bears a fluorescein group and possesses high-affinity sulfonamide-based moiety for CA IX recognition (*K*_d_, determined by FTSA of GZ19-32 binding to recombinant CA IX, equal to 10 pM). Simultaneous addition of the fluorescent compound and the test compound competed for the same CA IX binding sites on the cell surface in a dose-dependent manner. Affinities of the tested compounds matched those determined for purified CA IX enzymes (Matulienė *et al.*, 2022 ▸). Thus, the assay recognized CA IX and not other isozymes that may also be expressed on the cell surface, especially CA XII and CA XIV.

The competition assay was also helpful in following the expression of CA IX, its concentration, and amount dependence on the time of expression. No CA IX was detectable after 24-hour expression, but the numbers increased over the next three days. The number of CA IX residues exceeded one million per cell, mostly visible at the invadopodia, where the invasion of adjacent tissues is orchestrated. The concentration of CA IX reached ≈10 nM. Fig. 3 ▸(*d*) shows the VD10-13 compound dosing curve while keeping the concentration of the fluorescein-labeled compound constant (10 nM). Higher concentrations of the test compound out-competed the fluorescent compound. The midpoint of the dosing curve does not match the *K*_d_ but is approximately 100-fold higher because the midpoint here does not match the 50%-bound value.

The competitive binding assay confirmed that our designed compounds possessed extremely high picomolar affinity, as determined by the TSA (Matulienė *et al.*, 2022 ▸). Furthermore, the assay demonstrated that the compounds selectively recognized CA IX in the presence of other CA isozymes. Therefore, the compounds possessed high affinity and selectivity for CA IX.

### 2.3. Structure–thermodynamics correlations of protein–ligand binding

After crystal structural characterization of compound binding and thermodynamic measurements by the assays described above, we attempted to perform two kinds of correlations. First, we correlated compound chemical structural features, such as the presence or position of various functional groups, with the thermodynamic parameters of binding. Second, we correlated the compound–CA isozyme complex crystal structures with the thermodynamics of the compound binding to the protein. Both correlations are essential to fully understand the underlying forces that hold the complex together.

**Figure 4:**
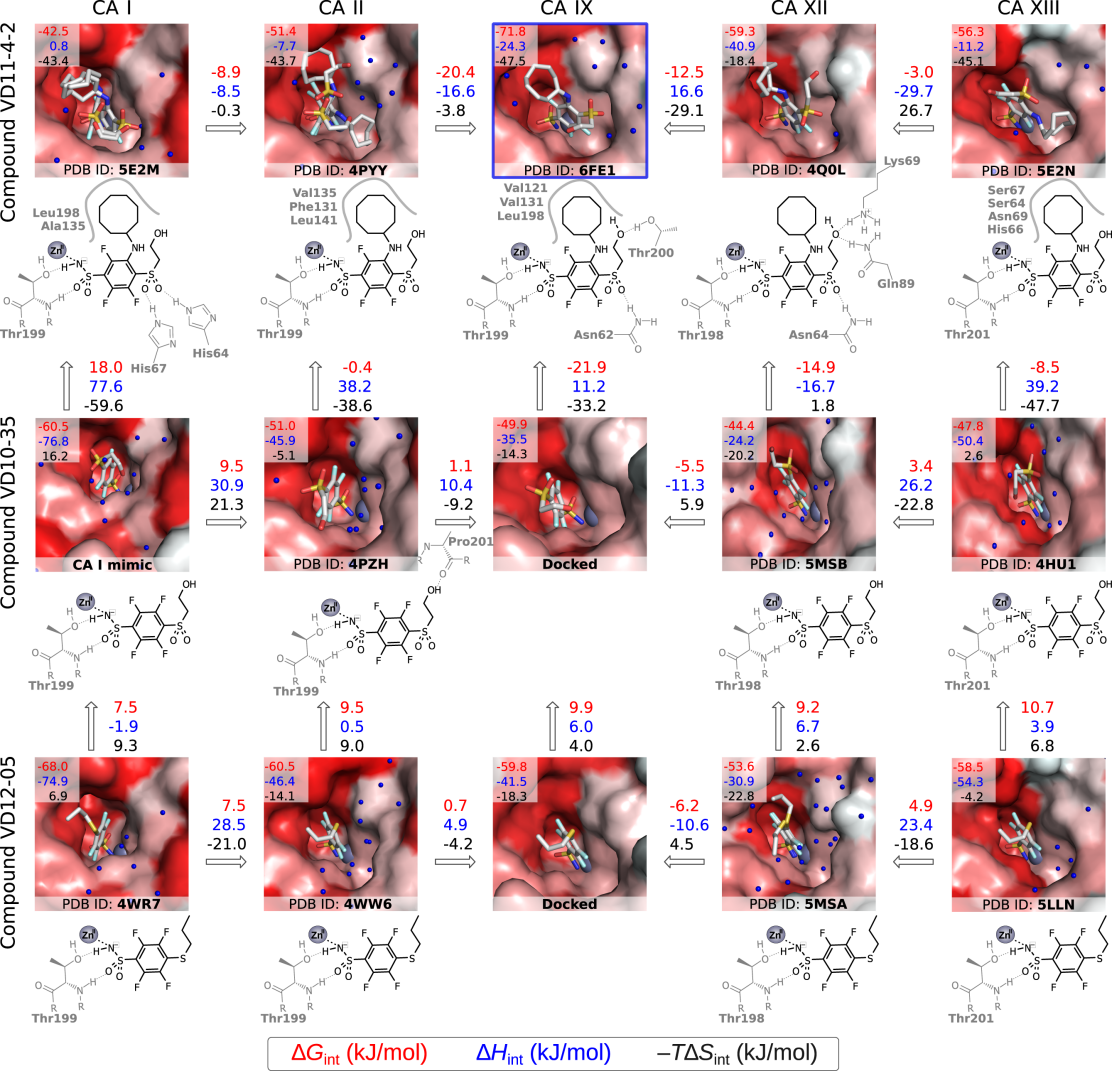
A map of correlation between three-dimensional X-ray crystallographic structure of enzyme–inhibitor complexes and binding thermodynamics. The three rows represent three compounds (VD11-4-2, VD10-35 and VD12-05) while the five columns represent CA isozymes (CA I, CA II, CA IX, CA XII and CA XIII). The three numbers on the upper-left corner of each crystal structure list the intrinsic standard Gibbs energy, enthalpy and negative entropy (multiplied by the absolute temperature) change upon binding to the CA isozyme (at 37°C). The PDB ID is listed at the bottom of each structure (except for two unavailable structures that are docked models). Chemical structure diagrams below each structure show interactions in the crystal structures. Arrows connect neighboring structures, and the numbers next to each arrow show the energy difference between binding processes when comparing different isozymes (horizontal arrows) or compounds (vertical arrows). The map shows binding energies and suggests structural reasons behind the energy changes.

Fig. 4 ▸ shows both correlations – here we compare three compound (VD11-4-2, VD10-35 and VD12-05) binding modes to five CA isozymes (CA I, CA II, CA IX, CA XII and CA XIII). These 15 binding pairs were characterized crystallographically (except VD10-35 and VD12-05 binding to CA IX) and thermodynamically. Intrinsic thermodynamic parameters of all pairs were determined by TSA and ITC (for calculations we used Gibbs energy obtained by TSA and enthalpy from ITC measurements).

First, we analyzed the Gibbs energy contributions that indicate compound affinities and selectivities. Comparing compounds VD12-05 and VD10-35, where the first contains the methyl group on the tail while the second contains the hydroxy group capable of making a hydrogen bond with bulk water or the protein, we see that all five CA isozymes lost 7.5–10.7 kJ mol^−1^ of Gibbs energy when changing the methyl group to the hydroxy group. A loss of 6 kJ mol^−1^ is equivalent to a 10× loss in *K*_d_ value. This result indicates that the hydroxy group preferentially made the hydrogen bond with water molecules not the protein molecule. Instead, the hydrophobic methyl group probably bound preferentially to the protein through non-specific hydrophobic interaction.

Another example is the addition of a cyclooctyl amino ring to the *meta* position relative to the sulfonamide group (going from the middle line to the top line in the figure). Here, the situation is quite different from the first example. The addition of the ring decreased compound affinity for CA I (by 18.0 kJ mol^−1^, equivalent to a 1000× loss in *K*_d_ affinity), but gained significant affinity or Gibbs energy for CA IX, −21.9 kJ mol^−1^, or −14.9 kJ mol^−1^ for CA XII. The gain of −21.9 kJ mol^−1^ is equivalent to 4000× gain in affinity *K*_d_. Therefore, while the compound VD10-35 was a stronger binder of CA I than of CA IX, the VD11-4-2 compound is a more than 100 000× stronger binder of CA IX than of CA I. Thus, the compound is highly selective for CA IX over CA I.

Second, we analyzed the enthalpies of interaction, shown as blue values in Fig. 4 ▸, and see no correlation between the Gibbs energies and the enthalpies of binding. The enthalpies show information on the contact between the compound and the protein. In contrast, the Gibbs energies show the sum of enthalpies and entropies (which provide information about the degrees of freedom in the free and bound states). Enthalpically, most exothermic reaction was for VD10-35 binding (−76.8 kJ mol^−1^) and VD12-05 binding (−74.9 kJ mol^−1^) to CA I. Both these reactions were driven by enthalpy with a minor opposition by entropy. In contrast, the VD11-4-2 compound binding to CA IX was largely driven by entropy (+47.5 kJ mol^−1^) and to a lesser extent by enthalpy (−24.3 kJ mol^−1^). Such balance of these forces led to the strongest binder (Gibbs energy of −71.8 kJ mol^−1^).

Schemes below the crystal structures show crystallographically observed interactions, hydrogen bonds and potential hydrophobic interactions. However, the exact assignment of the energetic contributions to every interatomic contact is still impossible, primarily due to a limited understanding of the thermodynamics and structure of a compound while free in solution.

**Figure 5:**
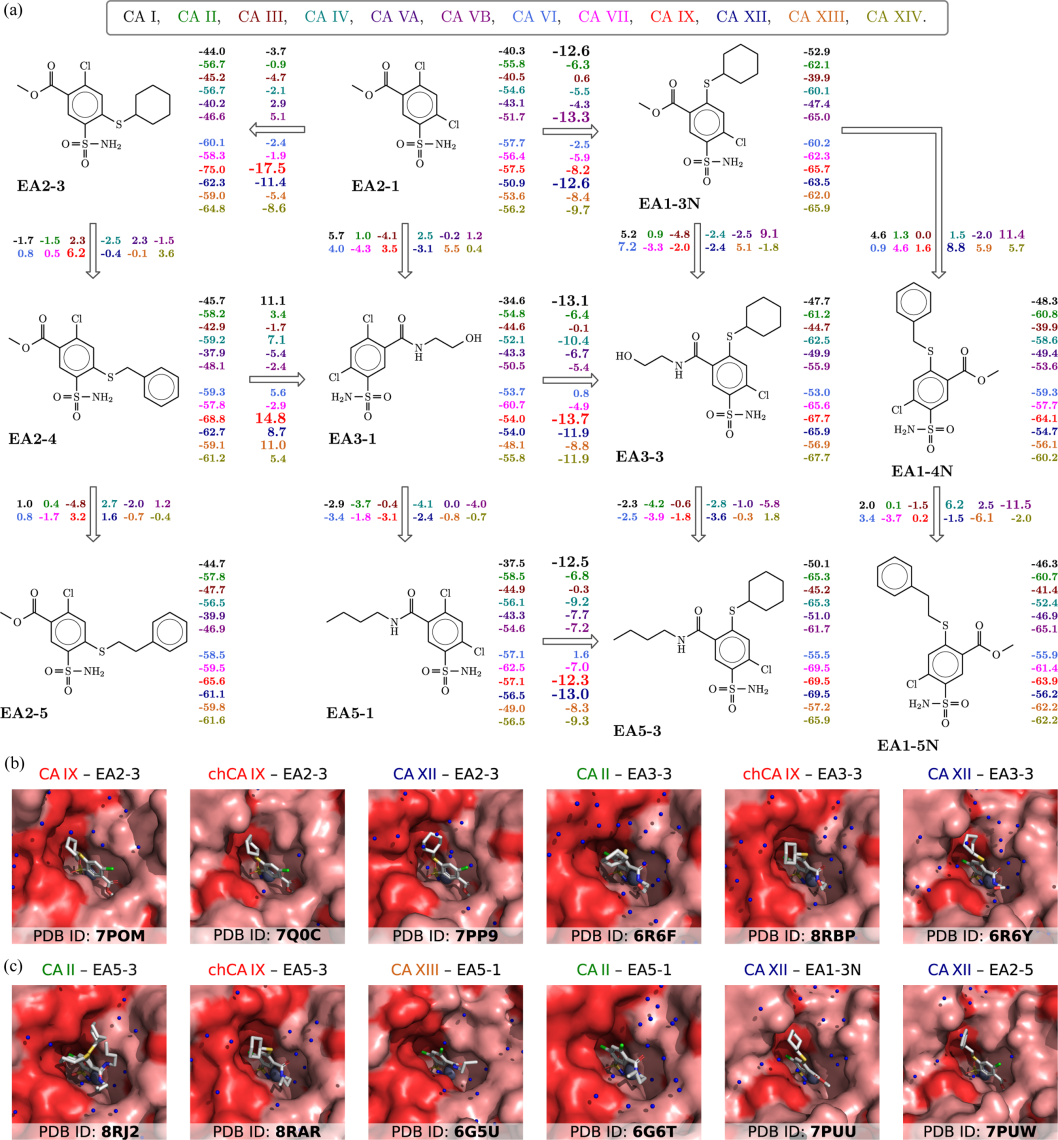
(*a*) A map of correlation between the chemical structures of compounds and their intrinsic affinities (changes in standard Gibbs energies upon binding at 37°C). The 12 numbers next to each compound structure show the intrinsic standard Gibbs energies of binding to each catalytically active CA isozyme. The 12 numbers next to each arrow connecting structurally related compounds show differences in the intrinsic standard Gibbs energy between the compounds. The most significant gains (negative numbers) or losses (positive numbers) in affinity are listed in larger font. Panels (*b*) and (*c*) show available X-ray crystallographic structures for several compounds binding to several CA isozymes. The map shows the path toward the most potent binder of CA IX, compound EA2-3.

Fig. 5 ▸ shows another example of the structure–thermodynamics correlation map for a series of compounds that do not contain fluorine atoms but instead have chlorine atoms. Unlike the previous figure, we do not show information on the enthalpy and entropy of interaction here. Instead, we show the binding Gibbs energies to all 12 catalytically active human CA isozymes. Having affinities for all 12 CA isozymes makes it easy to judge whether the compound will possess significant selectivity toward one or several isozymes. Numbers next to the structures in Fig. 5 ▸ show affinities for isozymes, while the numbers next to the arrows show the differences between the affinities of adjacent compounds. Thus, we can judge a contribution in affinity due to the chemical change of the compound structure.

In this series of EA compounds, the most notable is the addition of the cyclohexane group to compound EA2-1, leading to compound EA2-3. The change in structure led to a gain in Gibbs energy for CA IX of −17.5 kJ mol^−1^, equivalent to nearly a 1000× gain in affinity. This gain was observed primarily in CA IX and to a lesser extent in CA XII. The EA2-3 compound exhibited a binding Gibbs energy of −75.0 kJ mol^−1^, the highest affinity among the studied compounds, reaching the femtomolar range. However, this number shows the intrinsic affinity and would not be observed experimentally by applying any technique. The observed affinity is always lower than the intrinsic one.

## 3. Methods

### 3.1. Chemical compound synthesis

EA compounds were synthesized and analytically characterized as described by Zakšauskas *et al.* (2020 ▸, 2022 ▸). The synthesis of the VD series of compounds was described by Dudutienė *et al.* (2013 ▸, 2014 ▸). The synthesis of the fluorescein-labeled compound GZ19-32 was described by Matulienė *et al.* (2022 ▸).

### 3.2. CA isozyme preparation

Recombinant human CAs were expressed in *Escherichia coli* [CA I, CA II, chCA IX (mutant CA II A65S, N67Q, I91L, F130V, V134L, L203A), CA III, CA IV, CA VA, CA VB, CA VII, CA XII, CA XIII, CA XIV], yeast [CA IX for crystallization experiments (Leitans *et al.*, 2015 ▸)] or mammalian cells (CA VI and CA IX used for binding affinity experiments). Protein expression and purification protocols are described in: CA I (Baranauskienė *et al.*, 2009 ▸); CA II (Cimmperman *et al.*, 2008 ▸); CA IV (Mickevičiūtė *et al.*, 2018 ▸); CA VI (Kazokaitė *et al.*, 2015 ▸); chCA IX (CA II A65S, N67Q, I91L, F130V, V134L, L203A) CA III, CA VA, CA VB, CA IX, CA XIV (Dudutienė *et al.*, 2014 ▸); CA VII, CA XIII (Sūdžius *et al.*, 2010 ▸); and CA XII (Jogaitė *et al.*, 2013 ▸). The protein stock solutions were stored at −80°C. The protein concentration was determined by UV absorption at 280 nm.

### 3.3. Crystallization and structure determination

Crystals of chimeric CA IX protein (mutant CA II A65S, N67Q, I91L, F130V, V134L, L203A) reproducing CA IX active sites were prepared like crystals of CA II by the sitting-drop method. The crystallization solution contained 0.2 M ammonium sulfate, 0.1 M sodium–bicine (pH 9.0) and 2 M sodium–malonate (pH 7.0). The protein was concentrated to 29 mg ml^−1^ and mixed with an equal volume of reservoir solution. Crystals were soaked with EA3-3 and EA5-3 inhibitors and added to the crystallization solution at a final concentration of 1 mM for several days. Diffraction data were collected at the P14 EMBL beamline at the PETRA III storage ring (DESY, Hamburg, Germany). Data reduction was performed by *XDS* (Kabsch, 2010 ▸). *COMBAT* v. 7.1.002, *SCALA* v. 3.3.22, *TRUNCATE* v. 7.1.002 and other *CCP4* v. 7.1.002 tools were used for data processing (Agirre *et al.*, 2023 ▸). The structure was solved by molecular replacement using *MOLREP* (Vagin & Teplyakov, 2010 ▸) with a protein chain of PDB ID 3hlj (Baranauskienė *et al.*, 2010 ▸) as an initial model. The model was refined by *REFMAC* v. 5.8.0258 (Murshudov *et al.*, 2011 ▸) and rebuilt using *Coot* v. 0.9 (Emsley *et al.*, 2010 ▸). Inhibitor models were created and minimized by *AVOGADRO* v. 1.2.0 (Hanwell *et al.*, 2012 ▸).

### 3.4. Competition model for the determination of VD10-13 compound *K*_d_ values for cell-expressed CA IX

HeLa cell culture was grown in 12-well plates under hypoxia (1% O_2_) or normoxia (21% O_2_) for 70–76 h. The medium was removed and then 200 µl of a two compounds mixture containing 10 nM of fluorescent compound GZ19-32 and different concentrations of ligand VD10-13 from 0 to 80 µM (by performing 12 serial twofold dilutions with the last sample concentration of 0 nM) in the FB medium was added and incubated at 37°C under normoxia in the CO_2_ incubator for 20 min. Then, the compound solution was removed, and the cells were washed three times for 1.5–2 min with 400 µl of PBS (phosphate-buffered saline) at room temperature. Then 180 µl of TrypLE express enzyme (ThermoFisher) was added to each well and incubated in the CO_2_ incubator for 5–10 min until the cells were detached from the surface. After the addition of 20 µl of the Defined Trypsin Inhibitor solution (ThermoFisher), cells were resuspended by pipetting, and 150 µl of the suspension from each well was transferred to black Thermo Scientific Nunc MicroWell 96-Well Optical-Bottom Plates for fluorescence and absorbance measurements. The fluorescence was recorded at 485 nm excitation and 520 nm emission wavelengths in the plate reader (Synergy HTX, BioTek) (Matulienė *et al.*, 2022 ▸).

A ligand binding to a protein in the presence of a competing compound was described by Wang (1995 ▸). The fraction of fluorescently labeled GZ19-32 (ligand B), α_B_, bound to CA IX in the presence of VD10-13 (ligand A) can be calculated using the following set of equations: 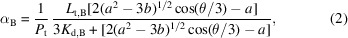

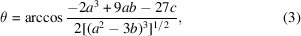

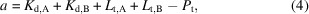

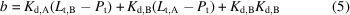
and
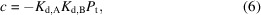
where *P*_t_ is the concentration of total added protein, and *L*_t,A_ and *L*_t,B_ are the molar concentrations of added ligands A and B, respectively. The *K*_d,A_ and *K*_d,B_ terms are the equilibrium dissociation constants of ligands A and B, respectively.

### 3.5. Stopped-flow assay of CO_2_ hydration

Recombinant human CA IX activity was determined by measuring the hydration rates of CO_2_ using an Applied Photophysics SX.18MV-R stopped-flow spectrometer. Reaction velocities were measured by recording the absorbance of pH indicator phenol red (final dye concentration of 30 µM) at 557 nm. The pH changes occur due to protons formed in the enzymatic or spontaneous CO_2_ hydration reaction. A saturated CO_2_ solution was prepared by bubbling CO_2_ in Milli-Q water at 25°C for 1 h. The samples consisted of 10 nM CA IX containing 0, 2.4, 9.8, 39, 156 or 625 nM inhibitor VD10-13 (final concentration of DMSO 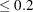
%) in 25 mM HEPES buffer containing 50 mM NaCl, pH 7.4. The *K*_d_ value was determined using the Morrison equation: 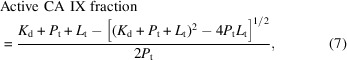
where *P*_t_ denotes the total concentration of CA IX, *L*_t_ is the total inhibitor VD10-13 concentration and *K*_d_ is the protein–ligand dissociation constant.

### 3.6. Estimation of binding affinity by fluorescent thermal-shift assay

5 µM CA IX solutions containing different concentrations of VD10-13 ranging from 0–200 µM (1.5-fold dilutions) and 100-fold diluted solvatochromic dye Glomelt (Biotium) were prepared in 50 mM sodium phosphate buffer (pH 7.0) containing 100 mM NaCl with a final 2%(*v*/*v*) concentration of DMSO. FTSA experiments were performed with real-time PCR instrument QIAGEN Rotor-Gene Q. The samples were heated from 25 to 95°C, increasing the temperature by 1°C min^−1^. Fluorescence measurements were taken at each temperature in the green channel (Ex/Em 470/510 nm). Protein-unfolding data analysis was performed, and compound binding constants were determined using the *Thermott* web server as described by Gedgaudas *et al.* (2022 ▸).

### 3.7. Estimation of binding affinity by isothermal titration calorimetry

100µM solution of the inhibitor VD10-13 or VD12-05 was prepared in 50 mM Tris buffer (pH 7.0) containing 100 mM NaCl (final DMSO concentration in solution 1%). A solution of 10 µM CA IX was prepared in 50 mM Tris buffer (pH 7.0) containing 100 mM NaCl and 1% DMSO solvent. The cell of the calorimeter PEAQ-ITC (Malvern Panalytical) was filled with the protein solution, and the syringe was filled with the inhibitor solution. The experiment was run at 37°C. Titrations consisted of a 1.2 µl first injection followed by 18 subsequent 2 µl additions every 150 s. Thermodynamic binding parameters were determined by fitting the data to a single binding-site mode using the *Microcal Origin* program (OriginLab, USA).

## 4. Discussion

Crystallography and other structural techniques, such as nuclear magnetic resonance or cryo-electron microscopy, have contributed significantly to structure-based drug discovery (Nasim & Qureshi, 2022 ▸). Insight into protein structure, especially of protein–lead compound complexes, could provide crucial information for successfully improving the designed compound, both in its increased affinity toward the disease target protein and its decreased affinity for all remaining proteins to avoid possible toxic side effects.

**Figure 6:**
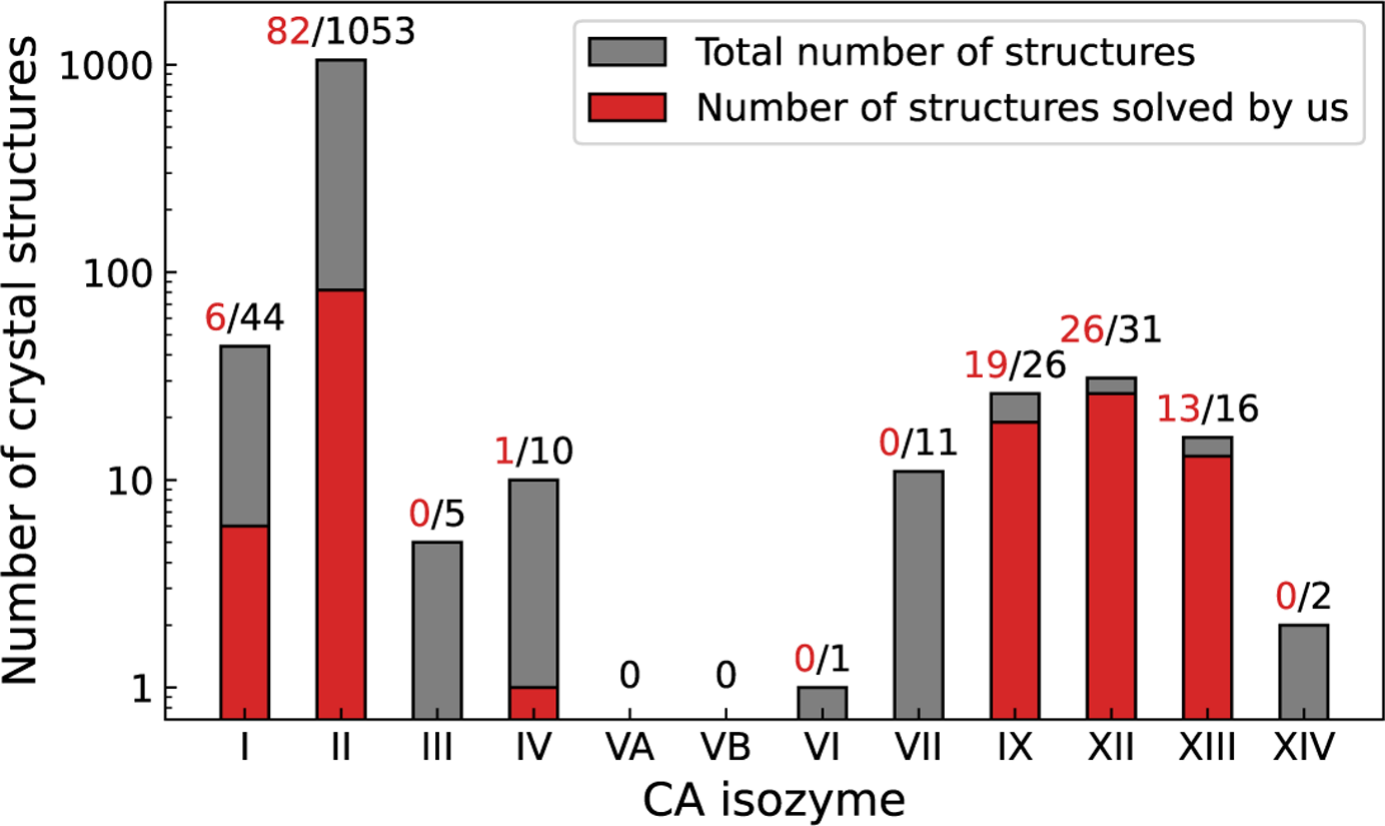
Numbers of X-ray crystallographic structures of catalytically active CA isozymes in the PDB. Red numbers show how many structures were solved and deposited by our research group. Most structures in the PDB are of CA II, a total of 1053, of which 82 are from our group. We have deposited a large part of the available structures of CA IX, CA XII and CA XIII. The availability of CA isozyme structures varies considerably; there are no structures available of both mitochondrial isozymes CA VA and CA VB, and only one structure is available of CA VI, two of CA XIV, and five of CA III. Values were obtained from the PDB on December 14, 2023.

The members of the CA family have been drug targets for a while. However, to this day, two human mitochondrial isozymes, CA VA and CA VB, still do not have crystal structures solved and deposited to the PDB. All remaining members of the catalytically active CA isozymes have their structures solved, but the numbers of solved structures are uneven for various isozymes. Over 1000 structures of CA II are available, but only one for CA VI. Our research group has significantly contributed to providing X-ray crystallographic structures of various CA isozymes bound with inhibitors (Fig. 6 ▸). We have deposited 147 structures to the PDB, especially a large share of CA IX, CA XII and CA XIII structures.

Crystallography has helped us significantly in the design of high-affinity compounds for CA IX, such as VD11-4-2 (Dudutienė *et al.*, 2014 ▸), GZ18-23 (Matulienė *et al.*, 2022 ▸) and EA2-3 (Zakšauskas *et al.*, 2022 ▸). These compounds possess affinities with *K*_d,obs_ in the range 20–300 pM. Furthermore, their selectivity toward CA IX over remaining isozymes reaches a hundred- to a million-fold depending on the isozyme. Thus, the compounds could be highly suitable for development as anticancer drugs that target CA IX. However, crystallography does not provide information on the compound affinity for proteins (Schlichting, 2005 ▸; Danley, 2006 ▸). Millimolar concentrations of inhibitors are used in crystallography. Thus, compounds possessing millimolar *K*_d_ would be visible in the structure and could be assumed as strong binders. In reality, such compounds could equally possess millimolar or picomolar *K*_d_ that would not be distinguishable by crystallography. Crystal structures often give an insight into the flexibility of inhibitors bound in the active sites (high *B* factors, partial occupancy, electron density, alternate conformations). Significant variability could be found in the position of ligands between several subunits.

Thermodynamic and kinetic assays must be performed to determine the binding between a purified protein and a pure compound. Various techniques are used for this purpose and have been extensively reviewed by Krishnamurthy *et al.* (2008 ▸). We have been using several orthogonal techniques, which are very important and significantly contribute to the overall picture of protein–ligand interaction.

The affinities are best determined by the TSA (Dudutienė *et al.*, 2020 ▸; Smirnovienė *et al.*, 2017 ▸). This assay is highly robust and easy to perform, and provides the most accurate affinities between stable globular proteins and synthetic small-molecule ligands (Linkuvienė *et al.*, 2018*b* ▸). This assay has the widest dynamic range for the *K*_d_ values, spanning from millimolar to picomolar. However, this assay has been underused, probably due to complex thermodynamic equations, which need to be solved for *K*_d_ determination. To ease this task, we have established a web-based service where a user can plug in their TSA data and obtain a suggestion of necessary thermodynamic parameters for the *K*_d_ determination. The freely available web application is called *Thermott* (Gedgaudas *et al.*, 2022 ▸).

Interaction kinetics is as important as thermodynamics. The on- and off-rates provide significant insight into the structural stability of the complex. We have also demonstrated that it is important to distinguish intrinsic from observed kinetics, similar to its importance in thermodynamics (Linkuvienė *et al.*, 2018*a* ▸,*b* ▸). ITC provided us with the enthalpies and the ITC measurements at several temperatures – the heat capacities of ligand binding, which have led us to a more complete understanding of the intrinsic binding principles (Linkuvienė *et al.*, 2018*b* ▸).

The enzyme-inhibition assay is the most abundant in the literature but is sometimes misused and misinterpreted. As explained in the literature (Copeland, 2013 ▸), such assays are unsuitable for tight-binding inhibitors and are limited to cases where the affinity of the inhibitor, *K*_d_, does not go below the enzyme concentration used in the assay. For CA inhibitors, where the affinity often goes to the picomolar range, it is not possible to distinguish very potent inhibitors and accurately determine the *K*_d_ value by the enzyme-inhibition assay.

It is insufficient to measure the affinity by one technique, and orthogonal assays should be used. Unfortunately, the use of a sole technique rather often leads to erroneous conclusions. We have stumbled upon this several times and suggest that others perform several independent assays. Therefore, since many of the measurements of CA IX potent inhibitors have been obtained solely by TSA, we searched for an independent method to confirm the affinity values and discovered that the competition model for binding to cell-expressed CA IX is an excellent technique to confirm binding of the compound to the protein (Matulienė *et al.*, 2022 ▸). This technique could be extended to other CA isozymes, especially those expressed on the cell membrane.

The abundance of data that contained numerous measurements between hundreds of compounds and 12 CA isozymes led to the establishment of a web-based database. The data were also scattered among numerous publications, and it took much work to compare them. Therefore, we compiled all data on the freely available web-based database (Lingė *et al.*, 2023 ▸). The database enables analysis of all accessible data in one place. There are quite a few databases available, but, to the best of our knowledge, our database is the only one that distinguishes intrinsic thermodynamics by dissecting linked protonation that accompanies most protein–ligand interactions. Dissection of these reactions, as explained in the *Introduction*[Sec sec1], led us to significantly different explanations of compound affinities and selectivities based on observed versus intrinsic data.

The concept of intrinsic parameters emphasizes the need to dissect all possible binding-linked reactions. These reactions could be protonation, but also could be linked to protein conformation, salt effects and many other reactions. To dissect these reactions, one needs to understand the binding mechanism. Subtraction of the binding-linked reactions would yield more accurate thermodynamic and kinetic binding parameters. These intrinsic parameters must be used in drug design to help rank compounds in their affinity, binding enthalpy, entropy or off-rate. However, the compound physiological effect should be evaluated according to the experimentally observed value. For example, the *K*_d_ value at pH 7.0 is not the intrinsic value, but the experimentally observed value will determine actual affinity at particular conditions because compound binding will be accompanied by all binding-linked reactions that will weaken the affinity. Therefore, the use of intrinsic parameters is not always necessary and depends on the task. Furthermore, in CA–sulfonamide binding, the mechanism involves two linked reactions, but in other protein–ligand binding cases, the mechanism might be quite different. Sometimes there may be no obvious linked reactions, but in our experience, there are more such reactions involved than appreciated by the scientific community.

While studying the family of CAs and their interaction with sulfonamide compounds, we have learned that the family is a convenient object to study general principles of compound–protein recognition, which is a still poorly understood phenomenon, and, therefore, underused in rational drug design. First, the CA isozymes have nearly identical folds, and the protein structures differ little in the shape of their active sites. Thus, studying the contribution of particular amino acids to the binding reaction is convenient. Second, the coordination bond between the sulfonamide nitrogen atom and the zinc of the protein strengthens the binding by more than 1000-fold. Thus, these compounds, even the simplest benzenesulfonamide, are strong binders of CA isozymes, which makes it easier to rationally synthesize a series of compounds where every compound will possess measurable binding affinity, enabling more efficient use of synthetic capabilities and more data for a deeper understanding of the recognition principles.

Despite abundant data, it is still rather discussable and controversial to state whether sulfonamide inhibitors bind and dynamically change the shape of the CA enzyme, as has been concluded by computational studies (Ma *et al.*, 2017 ▸), or whether the enzyme does not undergo substantial conformational changes upon ligand binding and the binding reaction is more like a ‘lock-and-key’ mechanism, as we have determined previously using crystallography (Dudutienė *et al.*, 2020 ▸). We think the truth lies between these extreme cases, and both mechanisms are possible.

The overall goal of rational drug design is to predict the chemical structure of a compound that would bind with great affinity to the disease-target protein and possess great selectivity by not binding to any of the 20 000+ proteins present in any cell to avoid possible toxic side effects. To achieve this goal, we must design a high-affinity compound *in silico* and demonstrate that the compound does not substantially bind to any other protein. This is not experimentally feasible but will probably be achieved by computer modeling in the future. When the design of such compounds is possible, the goal of rational drug design will be accomplished.

## Supplementary Material

PDB reference: chimeric human carbonic anhydrase IX with EA3-3, 8rbp

PDB reference: chimeric human carbonic anhydrase IX with EA5-3, 8rar

PDB reference: carbonic anhydrase II with EA5-3, 8rj2

## Figures and Tables

**Table 1:** X-ray crystallographic data-collection and refinement statistics All datasets were collected at 100 K and the test set size was 10%.

Isozyme–ligand	chCA IX–EA3-3	chCA IX–EA5-3	CA II–EA5-3
PDB ID	8rbp	8rar	8rj2
Data-collection statistics			
Space group	*P*2_1_	*P*2_1_	*P*2_1_
Unit-cell parameters (Å)	*a* = 42.12, *b* = 41.31, *c* = 72.12, α = γ = 90, β = 104.3	*a* = 42.13, *b* = 41.27, *c* = 72.40, α = γ = 90, β = 104.3	*a* = 42.07, *b* = 41.15, *c* = 72.03, α = γ = 90, β = 104.2
Resolution range (Å)	69.88–1.15	70.15–1.15	40.79–1.12
Wavelength (Å)	0.97552	0.97552	0.97552
Radiation source	EMBL beamline P14 at PETRA III storage ring (DESY, Hamburg, DE)	EMBL beamline P14 at PETRA III storage ring (DESY, Hamburg, DE)	EMBL beamline P14 at PETRA III storage ring (DESY, Hamburg, DE)
Unique reflections, overall (outer shell)	81171 (9330)	81933 (9111)	81344 (7994)
*R*_merge_, overall (outer shell)	0.049 (0.237)	0.118 (1.335)	0.046 (0.145)
〈*I*/σ〉, overall (outer shell)	17.4 (5.6)	5.8 (1.1)	25.1 (10.6)
Multiplicity, overall (outer shell)	6.5 (5.2)	6.5 (4.8)	6.9 (6.2)
Completeness (%), overall (outer shell)	95.1 (75.2)	94.7 (72.7)	89.3 (60.4)
Wilson *B* factor	10.863	10.741	9.087
			
Refinement statistics			
*R*_work_	0.11768	0.14114	0.14893
*R*_free_	0.14572	0.18011	0.17601
RMSD bond lengths (Å)	0.013	0.013	0.017
RMSD bond angles (°)	1.857	1.855	2.272
*B* factors			
Atoms in the structure			
All	19.99	21.97	17.58
Main chain	15.71	17.59	14.43
Side chain	20.44	23.18	17.88
Inhibitor	17.34	20.65	25.20
Waters	33.23	36.79	28.5
Zinc	8.45	10.3	7.27
Other molecules	31.05	26.13	29.36
Atoms in the structure (non-hydrogen)			
All	2583	2455	2459
Protein	2227	2189	2152
Inhibitor	24	25	25
Water	311	236	266
Zinc	1	1	1
Other molecules	20	4	15
Ramachandran statistics			
Favored regions (%)	95	97	97
Allowed regions (%)	5	3	3
Outliers	0	0	0
